# Supplementary material for: Nutritional Influences on Locomotive Syndrome
Source: J Clin Med. 2022 Jan 26;11(3):610. doi: 10.3390/jcm11030610 (PMC8836534; doi:10.3390/jcm11030610)
Supplement: Supplementary file 1 [file jcm-11-00610-s001.zip › jcm-1528121-supplementary.pdf]

Supplemental Table S1. The comparison of all parameters between non-elderly and elderly participants.

	All (n=368)	Adult (n=163)	Older Adult (n=205)	p
Male/Female	154/214	52/111	102/103	0.001*
Age(yrs)	63.8±10.5	54.3±7.3	71.3±5.3	<0.001*
Height(cm)	158.1±8.1	159.4±7.8	157±8.2	0.006*
Weight(kg)	59.2±11.5	59.6±12.3	58.9±10.8	0.553
BMI(kg/m2)	23.5±3.5	23.3±3.6	23.7±3.4	0.231
BFP (%)	29.1±6.6	29.8±6.3	28.5±6.8	0.057
SMI(kg/m2)	6.70±1.03	6.65±1.07	6.73±1.00	0.477
Grip strength(kg)	26.9±8.8	27.1±9.4	26.7±8.2	0.730
N/L	156/212	71/92	85/120	0.383
Hypertension(y/n)	140/228	31/132	109/96	<0.001*
Diabetes(y/n)	24/344	3/160	21/184	0.001*
Hypertension(y/n)	140/228	31/132	109/96	<0.001*
**Laboratory data**				
White blood cell(/μL)	5.8±1.5	5.7±1.5	5.8±1.6	0.506
Hemoglobin(g/dl)	13.5±1.2	13.4±1.3	13.6±1.1	0.175
Platelet(10^4/μL)	21.8±5.4	23±5.7	20.7±4.9	<0.001*
HbA1c(%)	5.7±0.5	5.5±0.4	5.8±0.5	<0.001*
Total Protein(g/dl)	7.2±0.4	7.2±0.4	7.3±0.3	0.178
Serum Albumin (g/dl)	4.4±0.2	4.4±0.2	4.3±0.2	0.183
ALP(U/L)	220.1±70.6	216.9±75.2	222.8±66.9	0.427
AST (U/L)	22.5±7.6	21.8±9.3	23±5.8	0.146
ALT((U/L)	22±12.2	22.7±14.9	21.4±9.4	0.308
γ-glutamyltranspeptidase(U/L)	31.2±34.7	35.1±46.5	28.1±20.6	0.055
Total-cholesterol(mg/dl)	207.2±33.2	212.6±31.8	202.9±33.7	0.005*
Triglyceride(mg/dl)	111.2±69.5	103.7±59.9	117.1±75.8	0.067
HDL-C(mg/dl)	61.5±14.9	62.9±14.5	60.4±15.1	0.103
LDL-C(mg/dl)	120.5±30.7	126.3±30.6	115.8±30	0.001*
Blood urea nitrogen(mg/dl)	14.7±5.2	12.8±3.4	16.1±5.9	<0.001*
Creatinine(mg/dl)	0.7±0.4	0.7±0.1	0.8±0.5	0.001*
Uric asid(mg/dl)	5.2±1.3	5.1±1.3	5.4±1.2	0.037*
Calcium(mg/dl)	9.2±0.3	9.2±0.3	9.2±0.3	0.726
C-reactive protein(mg/dl)	0.09±0.18	0.07±0.09	0.11±0.22	0.071
**Nutritional intake**				
Energy(kcal/day)	1644.1±389.5	1591.1±351.2	1686.3±413.4	0.020*
Protein(g/day)	53.3±13.7	51.5±10.8	54.8±15.6	0.022*
Fat(g/day)	44.6±13.5	42.9±12.2	46±14.3	0.031*
Carbohydrate(g/day)	229.1±69.5	221.1±67.6	235.4±70.6	0.050
Sodium(mg/day)	1972.3±689.4	1822.9±620.8	2091.1±719	<0.001*
Potassium(mg/day)	2152.7±577.7	2027.2±447.7	2252.5±647.1	<0.001*

Calcium(mg/day)	543.9±191.6	502.7±150.7	576.7±213.5	<0.001*
Iron(g/day)	7.0±2.3	6.5±1.8	7.4±2.6	<0.001*
Catotenes(mg/day)	3056.1±1589.6	2721±1165.4	3322.5±1818.4	<0.001*
VitaminA(μg/day)	855±587.3	821.2±665.3	881.9±517.2	0.325
VitaminD(μg/day)	7.3±3.9	6.5±3.2	7.9±4.3	0.001v
VitaminE(mg/day)	8.1±2.7	7.7±2.2	8.5±2.9	0.005*
VitaminB1(mg/day)	0.67±0.09	0.67±0.08	0.67±0.09	0.918
VitaminB2(mg/day)	1±0.3	1±0.2	1.1±0.3	0.001*
Folate(μg/day)	308.7±130.8	283.3±106.4	328.9±144.4	0.001*
VitaminC(mg/day)	87.5±40.5	76.6±26.2	96.2±47.3	<0.001*
SFA(g/day)	11.6±2.9	11.1±2.4	11.9±3.2	0.011*
MUFAd(g/day)	16.4±4.8	16.1±4.8	16.6±4.8	0.293
PUFA(g/day)	13.2±4.5	12.5±4.0	13.8±4.9	0.009*
Cholesterol(mg/day)	241.6±79.1	238.9±78.4	243.7±79.8	0.560
Soluble dietary fiber(g/day)	2.1±0.7	1.9±0.5	2.2±0.8	<0.001*
Insoluble dietary fiber(g/day)	8.1±2.7	7.3±1.7	8.7±3.1	<0.001*
Total dietary fiber(g/day)	11.4±3.8	10.3±2.8	12.3±4.3	<0.001*
n-3 PUFA(g/day)	2299.2±750.9	2195±764.7	2382±731	0.017*
n-6 PUFA(g/day)	11206.8±3901.2	10673.7±3523.9	11630.7±4136.5	0.019*
Energy from alcohol(kcal/day)	47.2±96.7	49±101.5	45.7±92.9	0.742
n-3 HUFAg/day)	754.6±403.1	682.2±344.5	812.1±436.5	0.002*

Valuse are expressed as means ± standard deviations
BMI: body mass index, BFP: body fat percentage, SMI: skeletal muscle mass Index, y/n: yes/no
SMI: skeletal muscle mass Index, N/L: normal group/ locomotive syndrome group, y/n: yes/no
HDL-C: High density Lipoprotein Cholesterol, LDL-C: Low Density Lipoprotein Cholesterol
SFA: saturated fatty acid, MUFA: Monounsaturated fatty acid, PUFA: Polyunsaturated fatty acid, HUFA: Highly-unsaturated fatty acid
All analyses are comparisons between adult and older adult.
The comparisons of categorial variables were done by chi-square test, and the comparisons of continuous variables were done by student t-test.
*:p<0.05

Supplemental Table S2. The comparison of all parameters between the N group and L group in adult participants.

	**Adult (n=163)**	**N (n=71)**	**L (n=92)**	**p**
Male/Female	52/111	28/43	24/68	0.090
Age(yrs)	54.3±7.3	53.2±8	55.1±6.7	0.094
BMI(kg/m2)	23.3±3.6	22.8±3.3	23.7±3.8	0.164
BFP (%)	29.8±6.3	28.2±5.1	31.0±6.8	0.005*
SMI(kg/m2)	6.65±1.07	6.72±1.04	6.60±1.09	0.489
Grip strength(kg)	27.1±9.4	29.6±9.8	25.1±8.7	0.002*
Hypertension(y/n)	31/132	9/62	22/70	0.075
Diabetes(y/n)	3/160	3/68	0/92	0.081
Hyperlipidemia(y/n)	27/136	11/60	16/76	0.833
**Laboratory data**				
White blood cell(/μL)	5.7±1.5	5.8±1.7	5.7±1.4	0.595
Hemoglobin(g/dl)	13.4±1.3	13.5±1.5	13.3±1.1	0.268
Platelet(10^{4}/μL)	23.0±5.7	23.0±6.3	23.1±5.3	0.910
HbA1c(%)	5.5±0.4	5.5±0.4	5.5±0.3	0.978
Total Protein(g/dl)	7.2±0.4	7.3±0.4	7.2±0.3	0.066
Serum Albumin (g/dl)	4.4±0.2	4.4±0.2	4.4±0.2	0.139
ALP(U/L)	216.9±75.2	215.1±77	218.2±74.1	0.800
AST (U/L)	21.8±9.3	21.4±7.6	22.2±10.5	0.624
ALT((U/L)	22.7±14.9	22.6±14.3	22.7±15.5	0.961
γ-glutamyltranspeptidase(U/L)	35.1±46.5	31.9±31.8	37.6±55.3	0.441
Total-cholesterol(mg/dl)	212.6±31.8	216.2±36.9	209.8±27.2	0.205
Triglyceride(mg/dl)	103.7±59.9	104.6±69.8	103.1±51.5	0.879
HDL-C(mg/dl)	62.9±14.5	61.8±14.7	63.8±14.4	0.397
LDL-C(mg/dl)	126.3±30.6	132.1±32.4	121.8±28.5	0.033
Blood urea nitrogen(mg/dl)	12.8±3.4	12.9±3.2	12.8±3.6	0.825
Creatinine(mg/dl)	0.7±0.1	0.7±0.1	0.6±0.1	0.224
Uric asid(mg/dl)	5.1±1.3	5.1±1.3	5.1±1.3	0.888
Calcium(mg/dl)	9.2±0.3	9.2±0.3	9.2±0.3	0.962
C-reactive protein(mg/dl)	0.07±0.09	0.07±0.08	0.08±0.1	0.509
**Nutritional intake**				
energy(kcal/day)	1591.1±351.2	1617.7±360.7	1570.6±344.4	0.397
protein(g/day)	51.5±10.8	51.3±9	51.7±12	0.808
fat(g/day)	42.9±12.2	42.0±9.2	43.6±14.1	0.408
carbohydrate(g/day)	221.1±67.6	228±71.9	215.7±63.9	0.251
Sodium(mg/day)	1822.9±620.8	1879.4±612.3	1779.3±627.2	0.309
Potassium(mg/day)	2027.2±447.7	2083.3±415.9	1983.9±468.5	0.161
Calcium(mg/day)	502.7±150.7	507.8±152.7	498.8±149.8	0.708
Iron(g/day)	6.5±1.8	6.4±1.8	6.5±1.9	0.689

Catotenes(mg/day)	2721±1165.4	2658.4±1130.7	2769.3±1195.3	0.549
VitaminA(μg/day)	821.2±665.3	732.9±296.2	889.3±842.4	0.137
VitaminD(μg/day)	6.5±3.2	6.1±2.5	6.8±3.6	0.138
VitaminE(mg/day)	7.7±2.2	7.5±1.6	7.9±2.6	0.247
VitaminB1(mg/day)	0.67±0.08	0.66±0.07	0.69±0.09	0.029
VitaminB2(mg/day)	1.0±0.2	0.9±0.2	1.0±0.2	0.160
Folate(μg/day)	283.3±106.4	268.3±88.6	294.9±117.6	0.115
VitaminC(mg/day)	76.6±26.2	73.4±24.4	79±27.4	0.178
SFA(g/day)	11.1±2.4	11.2±2.6	11.1±2.2	0.903
MUFAd(g/day)	16.1±4.8	15.5±3.3	16.5±5.7	0.170
PUFA(g/day)	12.5±4	12.3±2.9	12.7±4.6	0.571
Cholesterol(mg/day)	238.9±78.4	222.6±50.9	251.4±92.7	0.020*
Soluble dietary fiber(g/day)	1.9±0.5	1.9±0.5	1.8±0.6	0.878
Insoluble dietary fiber(g/day)	7.3±1.7	7.2±1.8	7.3±1.8	0.822
Total dietary fiber(g/day)	10.3±2.8	10.2±2.8	10.3±2.8	0.892
n-3 PUFA(g/day)	2195±764.7	2100.3±481.5	2268±922.2	0.166
n-6 PUFA(g/day)	10673.7±3523.9	10579.5±2506.8	10746.4±4154.5	0.765
Energy from alcohol(kcal/day)	49.0±101.5	39.2±67.3	56.6±121.4	0.280
n-3 HUFAg/day)	682.2±344.5	640±274.9	714.8±388.1	0.170

Valuse are expressed as means ± standard deviations. BMI: body mass index, BFP: body fat percentage, SMI: skeletal muscle mass Index, y/n: yes/no, ALP: alkaline phosphatase, AST: aspartate transaminase, ALT: alanine aminotransferase, HDL-C: High density Lipoprotein Cholesterol, LDL-C: Low Density Lipoprotein Cholesterol, SFA: saturated fatty acid, MUFA: Monounsaturated fatty acid, PUFA: Polyunsaturated fatty acid, HUFA: Highly-unsaturated fatty acid
All analyses are comparisons between N and L group.
The comparisons of categorial variables were done by chi-square test, and the comparisons of continuous variables were done by student t-test. *:$p<0.05$
There were significant differences in BFP, Grip strength, Gate speed, LDL-C, and Vitamin B1 between N and L group.

Supplemental Table S3. The comparison of all parameters between the N group and L group in older adult participants.

	Older Adult (n=205)	N (n=85)	L (n=120)	p
male/female	102/103	48/37	54/66	0.120
Age(yrs)	71.3±5.3	70.2±4.7	72.2±5.6	0.008*
BMI(kg/m2)	23.7±3.4	23.2±3.4	24.1±3.3	0.061
BFP (%)	28.5±6.8	26.7±6.3	29.7±6.9	0.002*
SMI(kg/m2)	6.73±1.00	6.86±1.06	6.64±0.95	0.140
grip strength(kg)	26.7±8.2	29.0±8.0	25.1±8.0	0.001*
Hypertension(y/n)	109/96	36/49	73/47	0.007*
Diabetes(y/n)	21/184	6/79	15/105	0.157
Hyperlipidemia(y/n)	81/124	36/49	45/75	0.305
**Laboratory data**				
White blood cell(/μL)	5.8±1.6	6.0±1.7	5.7±1.5	0.322
Hemoglobin(g/dl)	13.6±1.1	13.8±1.0	13.4±1.1	0.036*
Platelet(10^4/μL)	20.7±4.9	21.1±4.5	20.5±5.2	0.435
HbA1c(%)	5.8±0.5	5.8±0.4	5.8±0.5	0.496
Total Protein(g/dl)	7.3±0.3	7.3±0.3	7.2±0.4	0.581
Serum Albumin (g/dl)	4.3±0.2	4.4±0.2	4.3±0.2	0.030*
ALP(U/L)	222.8±66.9	230±79.7	217.7±55.8	0.195
AST (U/L)	23±5.8	23.3±5.9	22.8±5.8	0.494
ALT((U/L)	21.4±9.4	22.6±10.9	20.5±8.2	0.127
γ-glutamyltranspeptidase(U/L)	28.1±20.6	29.8±19.5	26.9±21.3	0.309
Total-cholesterol(mg/dl)	202.9±33.7	205.3±35	201.2±32.8	0.399
Triglyceride(mg/dl)	117.1±75.8	112.6±59.9	120.3±85.4	0.479
HDL-C(mg/dl)	60.4±15.1	61.1±15.3	59.8±15.0	0.55
LDL-C(mg/dl)	115.8±30	118.4±28.2	114±31.3	0.305
Blood urea nitrogen(mg/dl)	16.1±5.9	16.2±6.7	16.0±5.3	0.801
Creatinine(mg/dl)	0.8±0.5	0.8±0.6	0.8±0.4	0.452
Uric asid(mg/dl)	5.4±1.2	5.3±1.1	5.4±1.3	0.366
Calcium(mg/dl)	9.2±0.3	9.2±0.3	9.1±0.3	0.025*
C-reactive protein(mg/dl)	0.11±0.22	0.14±0.3	0.09±0.12	0.138
**Nutritional intake**				
energy(kcal/day)	1686.3±413.4	1638.8±348.7	1719.9±452	0.167
protein(g/day)	54.8±15.6	52.9±11.6	56.2±17.8	0.132
fat(g/day)	46±14.3	44±12.1	47.4±15.6	0.090
carbohydrate(g/day)	235.4±70.6	227.2±62	241.2±75.8	0.162
Sodium(mg/day)	2091.1±719	1961.1±586.7	2183.1±789	0.029*
Potassium(mg/day)	2252.5±647.1	2168.1±490.4	2312.2±734.4	0.117
Calcium(mg/day)	576.7±213.5	565.9±182.2	584.4±233.7	0.543
Iron(g/day)	7.4±2.6	7±1.9	7.7±2.9	0.059

catotenes(mg/day)	3322.5±1818.4	3101.9±1422.6	3478.8±2044.6	0.144
VitaminA(μg/day)	881.9±517.2	826.2±355.7	921.4±604.5	0.195
VitaminD(μg/day)	7.9±4.3	7.6±2.6	8.2±5.2	0.306
VitaminE(mg/day)	8.5±2.9	8.1±2.1	8.8±3.4	0.099
VitaminB1(mg/day)	0.67±0.09	0.66±0.08	0.68±0.10	0.114
VitaminB2(mg/day)	1.1±0.3	1.0±0.3	1.1±0.3	0.286
Folate(μg/day)	328.9±144.4	309.4±100.0	342.6±167.9	0.105
VitaminC(mg/day)	96.2±47.3	88.7±37.9	101.6±52.4	0.054
SFA(g/day)	11.9±3.2	11.8±3.1	12.0±3.3	0.720
MUFAd(g/day)	16.6±4.8	15.8±3.8	17.2±5.4	0.047*
PUFA(g/day)	13.8±4.9	13.0±3.3	14.3±5.7	0.063
Cholesterol(mg/day)	243.7±79.8	234.4±64.3	250.3±88.9	0.161
Soluble dietary fiber(g/day)	2.2±0.8	2.1±0.7	2.3±0.9	0.063
Insoluble dietary fiber(g/day)	8.7±3.1	8.2±2.4	9.1±3.5	0.065
Total dietary fiber(g/day)	12.3±4.3	11.6±3.5	12.8±4.8	0.050
n-3 PUFA(g/day)	2382±731	2271.3±518.1	2460.4±843.6	0.068
n-6 PUFA(g/day)	11630.7±4136.5	10898.3±2808.4	12149.4±4807.4	0.033*
Energy from alcohol(kcal/day)	45.7±92.9	51.9±87.2	41.3±96.9	0.426
n-3 HUFAg/day)	812.1±436.5	781±274.1	834.1±522	0.393

Value are expressed as means ± standard deviations. BMI: body mass index, BFP: body fat percentage, SMI: skeletal muscle mass Index, y/n: yes/no, ALP: alkaline phosphatase, AST: aspartate transaminase, ALT: alanine aminotransferase, HDL-C: High density Lipoprotein Cholesterol, LDL-C: Low Density Lipoprotein Cholesterol, SFA: saturated fatty acid, MUFA: Monounsaturated fatty acid, PUFA: Polyunsaturated fatty acid, HUFA: Highly-unsaturated fatty acid
All analyses are comparisons between N and L group.
The comparisons of categorial variables were done by chi-square test, and the comparisons of continuous variables were done by student t-test. *:p<0.05
There were significant differences in BFP, Grip strength, Gate speed, LDL-C, and Vitamin B1 between N and L group.
